# The mechanisms used by enteropathogenic *Escherichia coli* to control filopodia dynamics

**DOI:** 10.1111/j.1462-5822.2008.01254.x

**Published:** 2008-12-01

**Authors:** Cedric N Berger, Valerie F Crepin, Mark A Jepson, Ana Arbeloa, Gad Frankel

**Affiliations:** 1 Centre for Molecular Microbiology and Infection, Division of Cell and Molecular Biology, Imperial College LondonLondon SW7 2AZ, UK; 2 Department of Biochemistry, School of Medical Sciences, University of BristolBristol BS8 1TD, UK

## Abstract

Enteropathogenic *Escherichia coli* (EPEC) subverts actin dynamics in eukaryotic cells by injecting effector proteins via a type III secretion system. First, WxxxE effector Map triggers transient formation of filopodia. Then, following recovery from the filopodial signals, EPEC triggers robust actin polymerization via a signalling complex comprising Tir and the adaptor proteins Nck. In this paper we show that Map triggers filopodia formation by activating Cdc42; expression of dominant-negative Cdc42 or knock-down of Cdc42 by siRNA impaired filopodia formation. In addition, Map binds PDZ1 of NHERF1. We show that Map–NHERF1 interaction is needed for filopodia stabilization in a process involving ezrin and the RhoA/ROCK cascade; expression of dominant-negative ezrin and RhoA or siRNA knock-down of RhoA lead to rapid elimination of filopodia. Moreover, we show that formation of the Tir-Nck signalling complex leads to filopodia withdrawal. Recovery from the filopodial signals requires phosphorylation of a Tir tyrosine (Y474) residue and actin polymerization pathway as both infection of cells with EPEC expressing TirY474S or infection of Nck knockout cells with wild-type EPEC resulted in persistence of filopodia. These results show that EPEC effectors modulate actin dynamics by temporal subverting the Rho GTPases and other actin polymerization pathways for the benefit of the adherent pathogen.

## Introduction

The Rho GTPases are ubiquitous proteins expressed in yeast, plants and mammals. To date, at least 25 Rho GTPases have been identified in human cells where they regulate various cellular processes including actin polymerization, microtubule dynamics, cell cycle and transcriptional regulation, morphogenesis and migration (Etienne-Manneville and Hall, 2002). Among the Rho GTPases, Cdc42, RhoA and Rac-1 are particularly well characterized. The Rho GTPase Cdc42 is localized at the plasma membrane and Golgi network and induces formation of filopodia, regulates Golgi to endoplasmic reticulum transport, as well as endocytosis and exocytosis. RhoA, which is found at the plasma membrane and in the cytosol, promotes formation of stress fibres and focal adhesions, regulating cell shape, attachment and motility. Rac-1, which is found exclusively at the plasma membrane, stimulates formation of lamellipodia and membranes ruffles (Wennerberg and Der, 2004; Ridley, 2006).

The Rho GTPases act as molecular switches cycling between GTP-bound (active) and GDP-bound (inactive) conformations. Switching a GTPase on and off is mediated by guanine nucleotide exchange factors (GEFs) and GTPase activating proteins (GAPs) respectively (Rossman *et al*., 2005). The Rho GTPases transmit signals in a GTP-dependent manner by activating and/or recruiting downstream effector proteins to their sites of action. In addition, the GDP-bound Rho GTPases can interact with the Rho guanine nucleotide dissociation inhibitors (GDI) that prevent membrane association by masking the prenyl group and stabilize the inactive form. Because Rho GTPases play multiple roles in the cell and actively control actin cytoskeleton, they are common target of many microbial pathogens (Finlay, 2005). Indeed, a large number of toxins (Lemonnier *et al*., 2007) and bacterial effectors (Schlumberger and Hardt, 2005) regulate the Rho GTPases to allow bacteria or viruses (Favoreel *et al*., 2007) to invade, spread or survive in the eukaryotic cell. Among these bacteria are *Salmonella enterica* and *Shigella* spp. that invade eukaryotic cells and enteropathogenic *Escherichia coli* (EPEC), enterohemorrhagic *E. coli* (EHEC) and the mouse pathogen *Citrobacter rodentium*, which colonize the gut via attaching and effacing (A/E) lesion formation (Frankel and Phillips, 2008). The ability to induce A/E lesion is dependent on the LEE pathogenicity island that encodes gene regulators, the outer membrane adhesin intimin, a type III secretion system (T3SS) and several effector proteins that upon translocation are able to subvert diverse cellular functions (reviewed in Garmendia *et al*., 2005).

Recently, a number of known T3SS effector proteins from different bacterial pathogens were grouped together based on the presence of a conserved Trp-xxx-Glu (WxxxE) motif and their capacity to affect the Rho GTPase signalling pathways (Alto *et al*., 2006). The WxxxE effectors are found in *Salmonella* spp. (SifA and SifB), *Shigella* spp. (IpgB1 and IpgB2), EPEC strain E2348/69 (Map), EPEC strain B171 (Map, TrcA, EspM1), EHEC O157:H7 (Map, EspM1, EspM2) and *C. rodentium* (Map, EspM2, EspM3, EspT) (Arbeloa *et al*., 2008; Bulgin *et al.*, 2008).

The LEE-encoded Map (mitochondrial associated protein) was first described as an EPEC effector protein that is targeted to the mitochondria (Kenny and Jepson, 2000) via a small peptide signal corresponding to the amino terminal 44 amino acids. In yeast, Map enters the mitochondria after binding the mitochondrial translocase Tom22, Tom40 and the matrix chaperon mtHsp70 (Papatheodorou *et al*., 2006). Moreover, Map alters mitochondrial morphology and membrane potential *in vitro* and *in vivo* (Ma *et al*., 2006; Papatheodorou *et al*., 2006). In addition, Map triggers transient formation of filopodia in cultured human cell lines (Kenny *et al*., 2002; Alto *et al*., 2006). Kenny *et al*. (2002) showed that Map activates Cdc42, while others have suggested that Map can bypass Cdc42, triggering filopodia via a Cdc42 molecular mimicry mechanism (Alto *et al*., 2006).

Recently, Map was shown to bind, via its carboxy terminal PDZ ligand motif TRL, PDZ1 of the scaffold protein sodium/hydrogen exchanger regulatory factor-1 (NHERF1) (Alto *et al*., 2006; Simpson *et al*., 2006). Importantly, no filopodia were observed at 30 min post infection of HeLa cells with EPEC expressing truncated Map (MapΔTRL). The mechanism through which the Map-NHERF1 protein complex contributes to filopodia formation is not known. The aim of this study was to investigate the mechanism-governing Map-induced filopodia formation.

## Results

### Kinetic of filopodia formation on fibroblast 3T3 Swiss cells

**Fig. 1:**
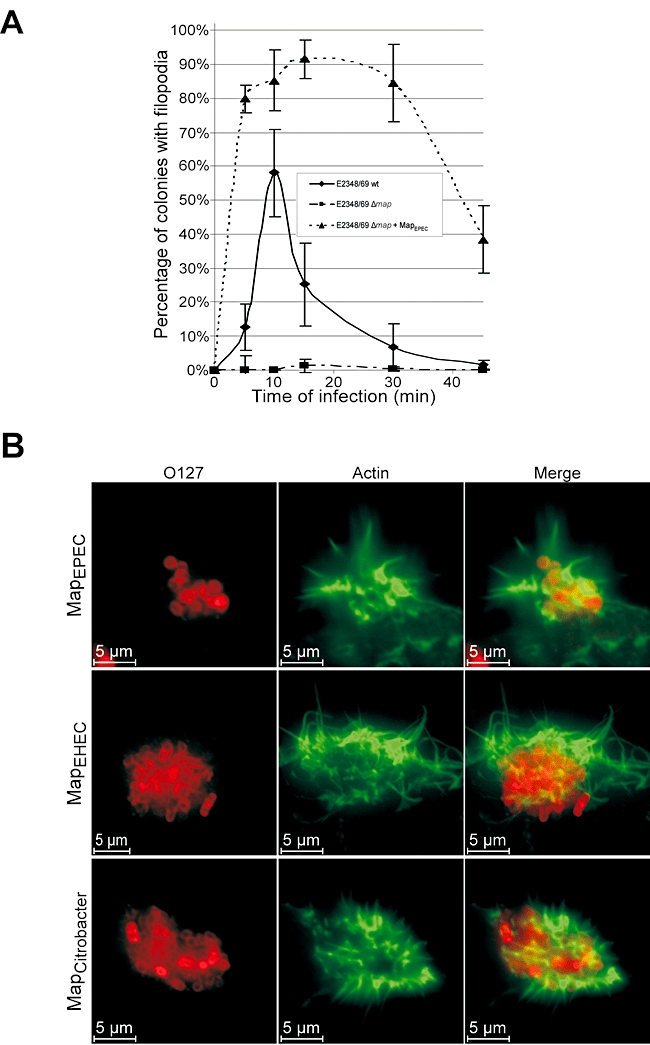
Kinetic of filopodia formation on 3T3 Swiss cells. A. Quantification of microcolony-associated filopodia on cell infected with wild-type E2348/69, E2348/69Δ*map* and E2348/69Δ*map* overexpressing Map_EPEC_. One hundred cells were counted in five independent experiments. Results are presented as mean ± SD. B. Fluorescence microscopy of 3T3 cells infected for 15 min with E2348/69Δ*map* overexpressing Map from EPEC, EHEC or *C. rodentium*. Actin was stained with Oregon green phalloidin (Green) and EPEC were detected with rabbit anti-0127 antibody (Red).

In order to investigate the mechanism implicated in Map-induced filopodia formation and withdrawal, we established a Swiss 3T3 cell infection model, as these cells exhibit more active actin dynamic than most cell lines. To characterize the kinetic of filopodia formation 3T3 Swiss cells were infected with wild-type EPEC strain E2348/69, E2348/69Δ*map* and *trans*-complemented E2348/69Δ*map*. Formation of Map-induced filopodia was recorded over time. Filopodia, associated with microcolonies (more than 10 bacteria), were first visible at 5 min post infection with wild-type EPEC, peaked at 10 min (seen in 58% of adherent microcolonies) and disappeared at 20 min (Fig. 1A). In contrast, filopodia were very rarely observed at any time point after infection with E2348/69Δ*map*. Overexpression of Map_EPEC_ significantly increased the number of microcolonies with filopodia (90%), which remained on the cell surface for more then 45 min. Similar results were obtained when Map_EHEC_ or Map_*CR*_ (*C. rodentium*) were expressed in E2348/69Δ*map* (Fig. 1B). Due to the fact that filopodia stayed for a longer period of time on cell infected with E2348/69Δ*map* overexpressing Map_EPEC_, we used this strain to dissect the signalling pathways involved in filopodia formation and persistence.

Substitution of the tryptophan (W74) and glutamine (E78) of the WxxxE motif with alanine abolished the capacity of Map_EPEC_ to induce filopodia (data not shown), confirming the original data of Alto *et al*. (2006). Immunofluorescence staining of Map_EPEC_ or Map_W74A/E78A_ showed that both proteins are targeted to the mitochondria, suggesting that the mutation did not affect cellular localization (data not shown). We employed site-directed mutagenesis to substitute other conserved residues (F82A, S84G, R85A, H133A, Y140A and C149A). This reveals that none of the single-amino-acid mutations affected filopodia formation (data not shown).

### Map-induced filopodia is Cdc42-dependent but Rac-1-independent

**Fig. 2:**
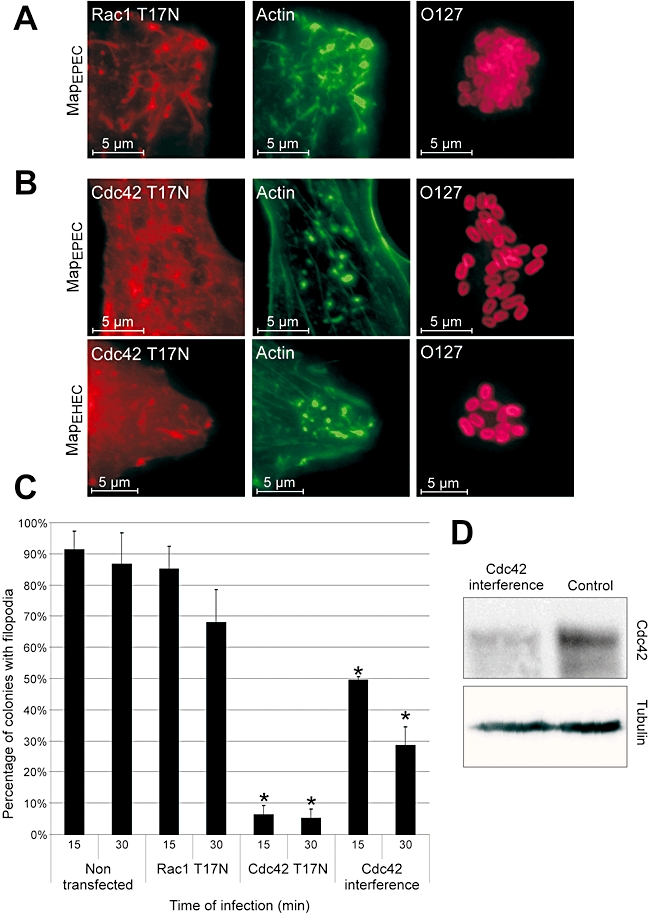
Filpodia formation by Map is Cdc42-dependent. 3T3 cells were transfected with dominant-negative Rac-1^T17N^ (A) or Cdc42^T17N^ (B) 24 h prior to infection with E2348/69Δ*map* overexpressing Map_EPEC_ or Map_EHEC_ for 15 min. Actin was stained with Oregon green phalloidin (Green), the Myc-tagged GTPases with mouse anti-myc (Red) and EPEC with rabbit anti-O127 (Magenta). Filopodia are observed on cells transfected with dominant-negative Rac-1 (A), but not on cells transfected with dominant-negative Cdc42 (B). Quantification of microcolony associated with filopodia in 3T3 cells transfected with dominant-negative Cdc42 or Rac-1 and Cdc42 siRNA (C). Cells were infected for 15 or 30 min with E2348/69Δ*map* overexpressing Map_EPEC_. One hundred colonies on transfected cell were counted in five independent experiments. Results are presented as mean ± SD. Significant differences from non-transfected cells are indicated by asterisks (*P < 0.01). Presence of filopodia induced by Map is affected by expression of Cdc42^T17N^ or Cdc42 siRNA, but not by expression of Rac-1^T17N^. The level of Cdc42 and Tubulin in cell lysates 48 h after transfection with Cdc42 siRNA was determined by Western blots (D).

To determine if Cdc42 is involved in Map-induced filopodia, 3T3 cells were transfected with dominant-negative Cdc42^T17N^; dominant-negative Rac-1^T17N^ was used as a control. Transfected cells were infected with E2348/69Δ*map* overexpressing Map_EPEC_ for 15 or 30 min. Immuno-staining of infected cells revealed no difference (*P*= 0.130) in the proportion of filopodia between mock-transfected cells or cells transfected with Rac-1^T17N^ (Fig. 2A and C). In contrast, transfection of Cdc42^T17N^ resulted in a complete inhibition of filopodia formation (Fig. 2B and C). Similar results were seen when transfected cells were infected with E2348/69Δ*map* overexpressing Map_EHEC_ (Fig. 2B). In order to confirm the role of Cdc42 in the filopodia formation process, small interfering RNA (siRNA) was used to knock down Cdc42; siRNA to knock down cortactin was used as a control. Western blot of cell lysates treated with control (data not shown) or Cdc42 siRNA was used to determine the knock-down efficiency (Fig. 2D). Although no complete Cdc42 knock-down was achieved (Fig. 2D), we observe a significant decrease in the number of filopodia on cells infected with E2348/69Δ*map* overexpressing Map_EPEC_ for 15 (50%) and 30 min (29%) (Fig. 2C), compare with the control siRNA-treated cells, which exhibited 81% and 73% filopodia respectively (data not shown).

### Map triggers Cdc42 activation

**Fig. 3:**
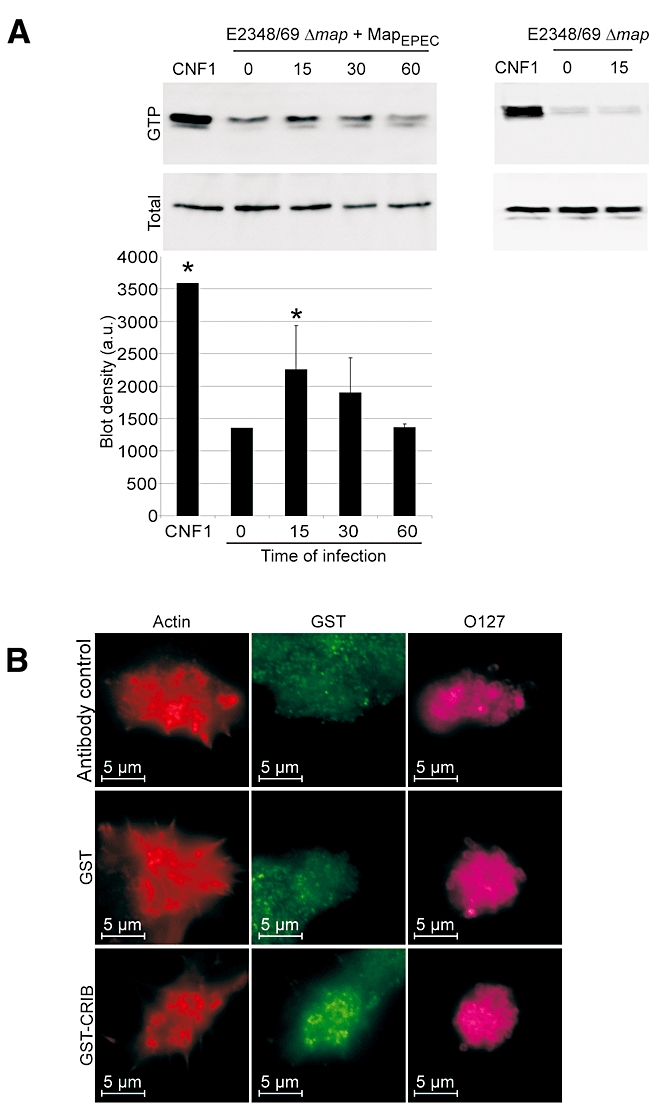
Map activates Cdc42. A. Swiss 3T3 cells were infected with E2348/69Δ*map* overexpressing Map_EPEC_ for 15, 30 or 60 min. CNF1 toxin was used as a positive control (25 μg ml^−1^). Cells were lysed and a GST–CRIB fusion protein was used to co-purify Cdc42-GTP. Total Cdc42 in the lysates and Cdc42-GTP were detected by Western blotting with anti-Cdc42 antibodies. The graph shows measurement of blot densities from three pull-down experiments (means ± SD). Significant differences from uninfected cells (time 0) are indicated by asterisks (*P < 0.05). Infection with E2348/69Δ*map* did not induce Cdc42 activation. B. Fluorescence microscopy of 3T3 cells infected for 15 min with E2348/69Δ*map* overexpressing Map_EPEC_. Fixed cells were pre-incubated with purified GST or GST–CRIB. Actin was stained with TRITC phalloidin (Red), GST and GST–CRIB were detected with rabbit anti-GST antibody (Green) and EPEC were detected with goat anti-*E. coli* antibody (Magenta). Specific signals, at the base of filopodia, were observed in cell treated with GST–CRIB whereas no signal could be detected for control antibody or GST alone.

In order to determine if Cdc42 is activated by Map_EPEC_ at the same time of filopodia formation, we specifically precipitated the active Cdc42-GTP form from extracts of 3T3 cells infected with E2348/69Δ*map* overexpressing Map_EPEC_ using a glutathione-S-transferase (GST)–CRIB of WASP fusion protein. CNF1 toxin, which activates all the Rho GTPases (Lemonnier *et al*., 2007), was used as a positive control (Fig. 3A). The ratio of activated and total Cdc42 was determined by densitometry. CNF1 treatment resulted in a 2.5-fold increase in Cdc42-GTP in comparison with uninfected cells. Infection with E2348/60Δ*map* overexpressing Map_EPEC_ resulted in c. twofold increase in the level of Cdc42-GTP at 15 and 30 min post infection compared with uninfected cells. In cells infected for 60 min, the level of activated Cdc42 was reverted to that of uninfected control cells (Fig. 3A). No Cdc42 activation was seen in cells infected with E2348/60Δ*map* 15 min post infection (Fig. 3A). These results show that activation of Cdc42 is Map-dependent and that the kinetic of Cdc42 activation parallels the kinetics of filopodia formation (Fig. 1A).

In order to localize activated Cdc42, 3T3 cells were infected with E2348/69Δ*map* overexpressing Map_EPEC_ for 15 min, fixed with PFA and permeabilized cells were incubated for 45 min with purified GST or GST–CRIB. Coverslips were processed for immunofluoresence using anti-GST antibodies. No specific signal was observed in untreated cells or cells incubated with GST alone (Fig. 3B). In contrast, we detected the GST–CRIB probe colocalized with the filopodia (Fig. 3B). We used pull-down and Western blot to confirm that GST–CRIB of WASP binds specifically to Cdc42 but not to Rac-1 or RhoA (data not shown).

### The PDZ-binding motif of Map is involved in stabilizing the Map-induced filopodia

**Fig. 4:**
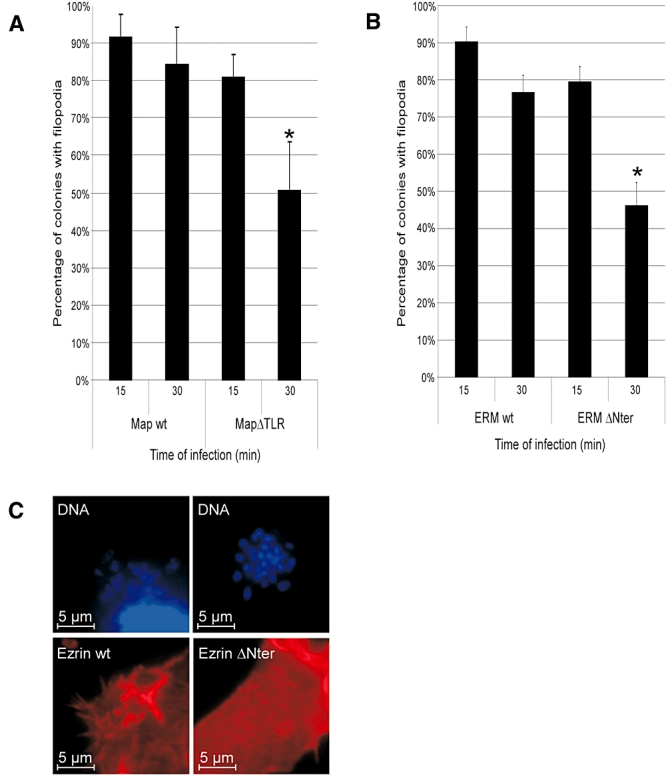
Filopodia stabilization is dependent on PDZ ligand motif (TRL) and ERM. Quantification of microcolony associated with filopodia on 3T3 cells (A) or 3T3 cell transfected with wild-type (wt) ezrin or dominant-negative ezrin (ΔNter) (B) infected for 15 or 30 min with E2348/69Δ*map* overexpressing Map_EPEC_ (A and B) or MapΔTRL (A). One hundred colonies were counted from five independent experiments. Results are presented as mean ± SD. Significant differences are indicated by asterisks (*P < 0.01). Expression of MapΔTRL had no effect at 15 min, but resulted in reduced filopodia at 30 min post infection (A). Map-induced filopodia is affected by expression of ezrin ΔNter at 30 min post infection (B). Wild-type ezrin, but not ezrin ΔNter, is recruited to the Map-induced filopodia (C); VSV-G-tagged ERM was stained with rabbit anti-VSV-G (Red) and DNA with Hoechst (Blue).

It was recently shown that Map, via its carboxy terminal PDZ ligand motif (TRL), binds NHERF1 (Alto *et al*., 2006; Simpson *et al*., 2006), which is recruited to the site of bacterial adhesion (Simpson *et al*., 2006). Alto *et al*. (2006) have shown that depleting cells of NHERF1 abolished Map-induced filopodia formation. In order to investigate how the NHERF1–Map protein complex might be involved in filopodia formation we infected 3T3 cells with E2348/69Δ*map* overexpressing wild-type Map_EPEC_ or MapΔTRL_EPEC_ for 15 and 30 min and the proportion of filopodia was determined microscopically. No significant difference in the proportion of filopodia was observed on cells infected with E2348/69 expressing wild-type or ΔTRL Map_EPEC_ at 15 min post infection (Fig. 4A). In contrast, by 30 min post infection with E2348/69 expressing MapΔTRL, there was a significant decrease in number of filopodia compared with cells infected with EPEC expressing wild-type Map (Fig. 4A).

NHERF1 is a scaffold protein containing a carboxy terminal ERM (ezrin, radixin and moesin) binding domain (Reczek *et al*., 1997). In order to determine if ezrin, which is colocalized with NHERF1 in Caco-2 cells infected with E2348/69 (Simpson *et al*., 2006), might have a role in stabilizing the filopodial structures, we transfected 3T3 cells with wild-type ezrin or with ezrin lacking the amino terminal domain (Algrain *et al*., 1993), which has a dominant negative activity. Transfected cells were infected with E2348/69Δ*map* overexpressing Map_EPEC_ for 15 or 30 min, processed for immunofluoresence and the filopodia associated with adherent microcolonies were enumerated. No significant difference (*P*= 0.056) was observed at 15 min post infection between cells transfected with wild-type or dominant-negative ezrin (Fig. 4B). However, a significant decrease (twofold) in the number of filopodia was observed on cell transfected with dominant-negative ezrin compared with control cells transfected with wild-type ezrin 30 min post infection. Localization studies (Fig. 4C) showed that wild-type ezrin was recruited to the filopodia at 15 min post infection; while diffuse cellular staining was observed in cell transfected with the dominant negative ezrin (Fig. 4C).

### RhoA/ROCK pathway is involved in stabilizing the Map-induced filopodia

**Fig. 5:**
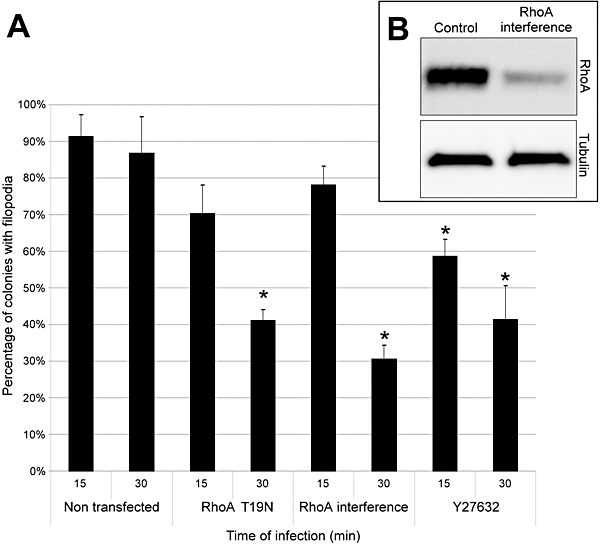
Filopodia stabilization is dependent on RhoA. A. Quantification of microcolony associated with filopodia on 3T3 cells transfected with dominant-negative RhoA^T19N^ and RhoA siRNA or pre-treated with ROCK inhibitor Y27632. Cells were infected for 15 or 30 min with E2348/69Δ*map* overexpressing Map_EPEC_. One hundred colonies on transfected cells were counted in five independent experiments. Results are presented as mean ± SD. Significant differences from non-transfected cells are indicated by asterisks (*P < 0.01). B. The level of RhoA and Tubulin in cell lysates 48 h after transfection with RhoA siRNA was determined on Western blots.

The ERM proteins can activate RhoA via the GEF Dbl (Lee *et al*., 2004; Martin-Villar *et al*., 2006) and the RhoA/ROCK (p160 Rho-associated coiled-coil containing protein kinase) complex is known to stabilize actin microfilaments by phosphorylation of cofilin (Arber *et al*., 1998). In order to determine if RhoA is involved in filopodia formation or stabilization, 3T3 Swiss cells were transfected with dominant-negative RhoA^T19N^ and filopodia was enumerated at 15 and 30 min post infection with E2348/69Δ*map* overexpressing Map_EPEC_ (Fig. 5A). No difference was recorded between mock-transfected cells or cells transfected with RhoA^T19N^ at 15 min post infection. However, at 30 min there was a twofold reduction in the number of microcolony-associated filopodia in cells transfected with RhoA^T19N^ (Fig. 5A). In order to confirm the role of RhoA, we transfected 3T3 Swiss cells with pRK5-myc-ROCK (aa 950–1069), which specially binds and sequesters RhoA. Filopodia were seen only after 15 min infection with E2348/69Δ*map* overexpressing Map_EPEC_ (data not shown).

In order to further confirm the role of RhoA in the filopodia stabilization process, siRNA was used to knock down RhoA. Western blot of cell lysates treated with control or RhoA siRNA showed that RhoA was efficiently knocked down (Fig. 5B). Infection of transfected cells with E2348/69Δ*map* overexpressing Map_EPEC_ showed that the number of filopodia was similar to that seen with the dominant-negative RhoA^T19N^, i.e. no significant difference in the number of filopodia at 15 min post infection and *c*. twofold reduction at 30 min (Fig. 5A).

ROCK is one of the main RhoA downstream effectors. To determine if Map activate the RhoA-ROCK pathway, Swiss 3T3 cells were incubated with the ROCK inhibitor Y-27632 for 1 h prior to infection with E2348/69Δ*map* overexpressing Map_EPEC_. Y-27632 is a highly specific inhibitor of ROCK-I and ROCK-II, which competitively excludes ATP from the catalytic site (Yamaguchi *et al*., 2006). Pre-incubation of Swiss 3T3 cells with Y-27632 significantly reduced the proportion of microcolonies with filopodia at 15 min (30%) and more drastically at 30 min (50%) post infection, These phenotypes parallel those seen with dominant-negative and siRNA RhoA (Fig. 5A).

Taken together, these results suggest that while formation of filopodia involves activation of Cdc42 by Map, stabilization of filopodia involves Map–NHERF1 protein interaction, ezrin and the RhoA-ROCK pathway.

### Activation of the Nck actin polymerization pathway is involved in filopodia withdrawal

**Fig. 6:**
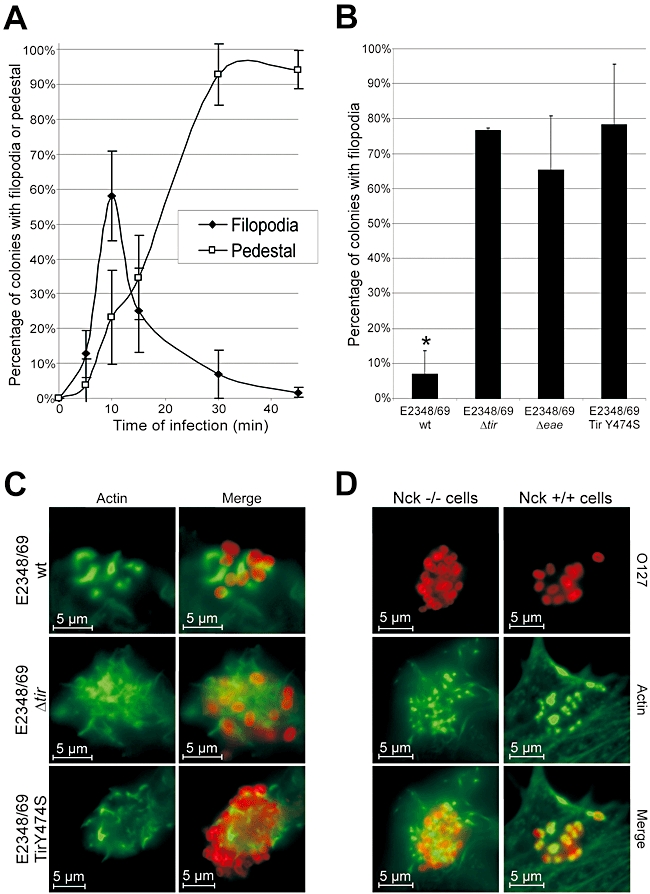
Downregulation of filopodia is dependent on Tir tyrosine phosphorylation and the Nck pathway. A. Quantification of microcolonies associated with filopodia or pedestals in 3T3 cell infected with wild-type E2348/69. One hundred cells were counted in five independent experiments. Results are presented as mean ± SD. B. Quantification of microcolonies associated with filopodia in cells infected for 30 min with E2348/69, E2348/69 Δ*tir*, E2348/69 Δ*eae* and E2348/69 expressing TirY474S. One hundred cells were counted in five independent experiments. Results are presented as mean ± SD. Significant differences from E2348/69 Δ*tir* are indicated by asterisks (*P < 0.01). No filopodia were observed on cells infected with wild-type E2348/69 while filopodia were present on cell infected with E2348/69 Δ*tir*, E2348/69 Δ*eae* and E2348/69 expressing TirY474S. C. Fluorescent microscopy of 3T3 cells infected for 30 min with E2348/69, E2348/69 Δ*tir* and E2348/69 TirY474S. Actin was stained with Oregon green phalloidin (Green) and EPEC were detected with rabbit anti-0127 antibody (Red). Pedestals are seen on cells infected with E2348/69 whereas filopodia are observed on cells infected with E2348/69 Δ*tir* and E2348/69 expressing TirY474S. D. Wild-type or Nck-deficient MEF cells were infected for 15 min with E2348/69 Δ*map* overexpressing Map_EPEC_. Actin was stained with TRITC phalloidin (Red) and EPEC were detected with rabbit anti-0127 antibody (Magenta). Filopodia are seen on infected Nck-deficient MEF cells whereas pedestals are observed on infected control Nck+/+ cells.

Filopodia formation after infection with wild-type E2348/69 is transient, lasting for only 30 min post infection (Fig. 6A). In parallel to filopodia withdrawal EPEC activates another actin polymerization branch involving binding of the outer membrane adhesin intimin to the T3SS effector Tir, which integrates into the plasma membrane of the host cell (Kenny *et al*., 1997). Binding of intimin leads to Tir clustering, Tir tyrosine phosphorylation, recruitment of Nck and activation of N-WASP leading to actin polymerization into pedestal-like structures under attached bacteria (reviewed in Garmendia *et al*., 2005). Infection of 3T3 cells has shown that pedestals start to appear 5 min post infection with wild-type E2348/69; the proportion of adherent bacteria associated with pedestal rise sharply thereafter, levelling at 30 min post infection, at the time when filopodia were already withdrawn (Fig. 6A).

In order to explore the mechanism of filopodia withdrawal, we infected 3T3 cells with E2348/69Δ*tir* and the proportion of filopodia was determined 30 min post infection. This revealed that deletion of *tir* resulted in constitutive, rather than transient, filopodia expression (Fig. 6A and B). In order to determine if Tir clustering was needed for Tir-mediated filopodia withdrawal, 3T3 cells were infected with E2348/69Δ*eae* lacking intimin. Immunofluorescence analysis has shown that in the absence of Tir clustering there was a constitutive expression of filopodia (Fig. 6B), confirming previous result of Kenny *et al*. (2002).

Clustering of Tir_EPEC_ by intimin leads to tyrosine 474 phosphorylation, recruitment of Nck and actin polymerization (reviewed by Caron *et al*., 2006). In order to determine if this cascade plays a role in Tir-mediated filopodia withdrawal we infected 3T3 cells with a chromosomal E2348/69 mutant expressing TirY474S that is unable to activate the Nck pathway (Schuller *et al*., 2007). Immunofluorescence analysis revealed constitutive expression of filopodia at 30 min post infection, which resembles the phenotype of E2348/69Δ*tir* (Fig. 6A and B). In order to further confirm the role of the Tir-Nck cascade in filopodia withdrawal, we infected Nck double knockout MEF cells with E2348/69 overexpressing Map, using wild-type MEF cells as a control. Unlike the kinetic of filopodia formation on Swiss cells, infection of the control Nck+/+ cells resulted in quick filopodia withdrawal and pedestal formation at 15 min post infection. In contrast, we observed extensive filopodia expression in the absence of pedestals on the Nck-deficient MEF cells (Fig. 6C). In order to confirm that Nck is the only protein involved in filopodia withdrawal, we transfected the knockout cells with GFP-Nck (using GFP alone as a control), which were infected with E2348/69 overexpressing Map_EPEC_ 18 h later. While filopodia were observed on cell transfected with GFP only, no filopodia were seen on cells transfected with GFP-Nck (data not shown). These results indicated that the Nck pathway plays a major role in filopodia withdrawal.

## Discussion

Rho GTPases are common targets of T3SS effectors (reviewed in Finlay, 2005). For example, the *Salmonella* Spi1 effector SopE functions as a GEF for Cdc42 and Rac-1 to enable bacterial internalization, before being targeted to the proteosome (Schlumberger and Hardt, 2005). Another Spi1 effector, SptP, then functions as a GAP turning the SopE signalling off. In this paper we have shown that EPEC also uses a two-component mechanism to modulate Rho GTPases; i.e. while Map activates Cdc42 and RhoA, inactivation involves the Tir-Nck-N-WASP signalling complex.

The cross-talk between Map and Tir requires biologically active effectors as EPEC overexpressing Map_W74A/E78A_ has the same phenotype as EPEC Δ*map* (i.e. microcolonies are exclusively associated with pedestals) while EPEC expressing TirY474S behaves as EPEC Δ*tir* (i.e. microcolonies are exclusively associated with filopodia). Consistent with this, inhibition of the Cdc42 pathway blocked filopodia formation and led to widespread pedestals at early time points post infection while eliminating Nck, which links Tir to the actin cytoskeleton, blocked pedestal formation and promoted a long-term presence of filopodia.

Infection studies using animal models have shown that Tir is an essential virulence factor as deletion of *tir* results in severe colonization attenuation (reviewed in Frankel and Phillips, 2008). In contrast, deletion of Map appears to have subtle effects, as in mixed infections of wild type and *map* mutant strains the latter is out-competed by the former (Mundy *et al*., 2004). The fact that filopodia are seen first suggests that the presence of active Cdc42 and RhoA prior to Tir-mediated actin polymerization is advantageous. Indeed, using human intestinal *in vitro* organ culture it was recently shown that an EPEC *map* mutant induces formation of ‘defective’ pedestals as the bacteria frequently fall off, leaving behind pedestal footprints (Shaw *et al*., 2005). How Map, Cdc42 and RhoA impact on the Tir-Nck-N-WASP signalling pathway is currently not known.

Map is a member of the WxxxE effectors (Alto *et al*., 2006), which include the *Shigella* IpgB1 and IpgB2, the *Salmonella* SifA and SifB and the EPEC and EHEC effectors Map, EspM and EspT (Alto *et al*., 2006; Arbeloa *et al*., 2008; Bulgin *et al.*, 2008). Alto *et al*. (2006) suggested that IpgB1 mimics Rac-1, IpgB2 mimics RhoA and Map mimics Cdc42 in a GTPase-independent mechanism. A recent publication by Handa *et al*. (2007) has shown that by binding the ELMO-DOCK180 GEF complex IpgB1 activates Rac-1. More recently, EspM effectors were shown to function upstream of RhoA activation leading to stress fibre formation (Arbeloa *et al*., 2008). In this study, we demonstrated that Map (of EPEC, EHEC and *C. rodentium*) translocation leads to activation of Cdc42 which colocalizes within the filopodia structures themselves; this result is in agreement with the mechanism proposed by Kenny and Jepson (2000). The different conclusions regarding Map-induced filopodia could be due to the fact that while Alto *et al*. (2006) used long-term (several hours) HEK293A cells culture cotransfected with Map and dominant-negative Cdc42, in Kenny *et al*. (2002) and in this study Map was delivered into HeLa and 3T3 cells, respectively, by infection and the phenotype was recorded only minutes later. The decisive role of the WxxxE motif was confirmed in both infection models (Alto *et al*., 2006). By mutating other conserved Map residues we could not identify any other essential single amino acids.

Alto *et al*. (2006) and Simpson *et al*. (2006) reported that the PDZ binding domain of Map is crucial for filopodia formation. These conclusions were made after infection or transfection of epithelial cells. In this study, using the highly dynamic 3T3 cells we found that while the PDZ-binding motif of Map plays no role in filopodia formation *per se*, it appears to have a role in retaining the formed filopodial structures on the cell surface. This activity is dependent on RhoA. One possible link between NHERF1 and RhoA is the ERM proteins, particularly ezrin. Indeed, an identical phenotype (i.e. rapid filopodia withdrawal with increased proportion of pedestals) was seen after infection of 3T3 cells with EPEC expressing MapΔTRL or infection of 3T3 cells transfected with dominant-negative RhoA or ezrin with E2348/69 Δ*map* overexpressing Map_EPEC_. Similar result was obtained after infection of 3T3 cells treated with RhoA siRNA.

**Fig. 7:**
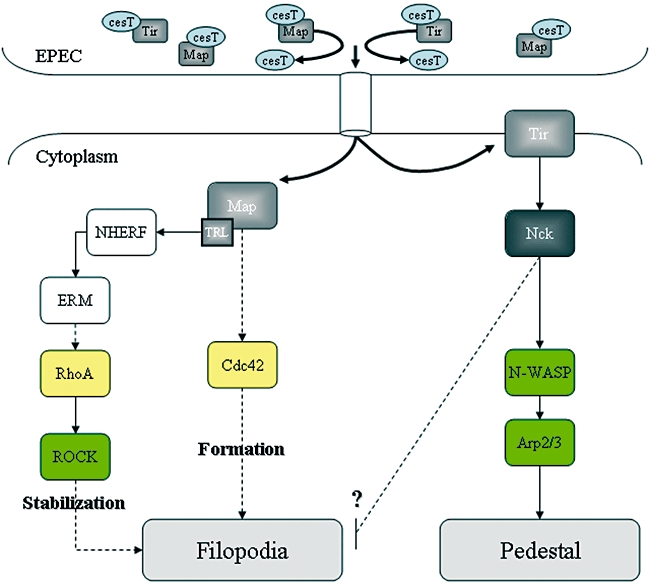
A model of the mechanism used by EPEC to trigger filopodia formation and withdrawal. Prior to translocation Map and Tir are maintained in a translocation competent confirmation by association with the CesT chaperone (Creasey *et al*., 2003). Once translocated Map sequentially activates Cdc42 and RhoA. Activation of the Tir-Nck actin polymerization pathway leads to recovery from the filopodial signals (see text for further details).

Based on our data we can now suggest a model (Fig. 7) in which EPEC infection triggers signal transduction pathways that while individually lead to either filopodia or pedestal formation, when crossed over fine-tune modulation of actin dynamic for the benefit of the adherent bacteria. According to our model following EPEC adhesion to the eukaryotic cell surface, either directly or via interaction with as yet unidentified GEF, Map activates Cdc42, leading to filopodia formation. Before clearing the cytosol and targeting the mitochondria, Map (alone or in complex with the GEF) transitorily interacts via its PDZ ligand motif with PDZ1 of NHERF1 (Alto *et al*., 2006; Simpson *et al*., 2006). This leads to recruitment of activated ezrin (Simonovic *et al*., 2001; Morales *et al*., 2004). Ezrin can then interact with GEF Dbl (Takahashi *et al*., 1998) or with Rho guanine nucleotide dissociation inhibitors (Takahashi *et al*., 1997), leading to activation of RhoA (Takahashi *et al*., 1997) and the RhoA-ROCK pathway, which stabilizes the actin microfilaments within the filopodia via phosphorylation of cofilin (Arber *et al*., 1998).

Tir is inactive before it has integrated into the eukaryotic cell plasma membrane and been clustered by intimin. This interaction induces tyrosine phosphorylation of Tir (Y474), recruitment of Nck and activation of N-WASP, leading to Arp2/3-mediated actin polymerization. Activation of this signal transduction pathway, probably in conjunction of Map mitochondria targeting, signals filopodia withdrawal. Downregulation of filopodia could be due to the fact that Nck sequesters N-WASP from the Cdc42-GTP pathway, as the former was shown to have a greater affinity to N-WASP than the latter (Tomasevic *et al*., 2007). Alternatively, Tir-bound Nck might trigger local activation of GC-GAPs (Zhao *et al*., 2003), which were reported to specifically inactivate Rac-1 and Cdc42. It is important to note that Kenny *et al*. (2002) have suggested that filopodia withdrawal from infected HeLa cells is due to a GAP ‘GXLR’ motif localized at the carboxy terminus of Tir itself.

In summary, our studies revealed a unique feedback regulation of actin dynamics by protein complexes and signal transduction pathways involving bacterial and eukaryotic proteins. The EPEC effectors appear to fine-tune temporal activation and de-activation of Rho GTPases and Nck recruitment for the benefit of the extracellular adherent bacteria. Elucidating this delicate process will undoubtedly contribute to a better understanding of EPEC disease, but will also likely lead to discovery of novel signalling pathway as the study of bacterial pathogens often does.

## Experimental procedures

### Bacterial strains and plasmids

The *E. coli* strains used in this study and their origin are listed in Table 1. Bacteria were cultured in Luria–Bertani (LB) broth at 37°C for 18 h with appropriate antibiotics. Overnight cultures were diluted to 1:100 in Dubelcco's minimal Eagle medium high glucose (4500 mg.l^−1^) and grown for 3 h at 37°C without agitation complemented, when needed, with 1 mM isopropyl-β-D-thiogalactopyranoside for 30 min.

**Table 1:** List of strains and plasmids.

	Description	Origin
Strain		
E2348/69	Wild-type EPEC 1 O127:H6	Levine *et al*. (1978)
E2348/69 Δ*map*	ICC202	Simpson *et al*. (2006)
E2348/69 Δ*tir*		This study
E2348/69 Tir Y474S		Schuller *et al*. (2007)
CVD206	E2348/69 Δ*eae*	Donnenberg and Kaper (1991)
Plasmids		
pSA10	pKK177-3 derivative containing *lac*^*I*^	Schlosser-Silverman *et al*. (2000)
pICC330	pSA10-*map* (EPEC O127:H6 E2348/69)	Simpson *et al*. (2006)
pICC331	pSA10-*map*_ΔTRL_	Simpson *et al*. (2006)
pICC386	pSA10-*map*_*AxxxA*_	This study
pICC387	pSA10-*map*_*F82A*_	This study
pICC388	pSA10-*map*_*S84G*_	This study
pICC389	pSA10-*map*_*R85A*_	This study
pICC390	pSA10-*map*_*Y140F*_	This study
pICC391	pSA10-*map*_*C149A*_	This study
pICC392	pSA10-*map*_*H133A*_	This study
pICC393	pSA10-*map* (EHEC O157:H7 sakai)	This study
pICC394	pACYC-*map* (*C. rodentium*)	This study

### Mutagenesis and plasmid generation

Primers used in this study are listed in Table 2. The genes encoding effector proteins were amplified by PCR using genomic DNA as template and cloned into pSA10. *map*_EHEC_ was amplified from EHEC O157:H7 strain EDL933, *map*_CR_ gene from *C. rodentium* strain ICC169.

**Table 2:** List of primers.

Names	Sequences	Restriction enzyme
Map W_74A/E78A_-Fw	5′-AAACAAGCGCAGATTACTTTTCTATCC-3′	PmeI
Map _W74A/E78A_-Rv	5′-AAACGCTTGCTGGGTATCACTACTACC-3′	
Map_F82A_-Fw	5′-CGCGAGCAGTAAACAAAACTGTGGATG-3′	NruI
Map_F82A_-Rv	5′-ATAGAGCAGTAATCTGCTCTTGCTTGAAC-3′	
Map_S84G_-Fw	5′-CGAGCAGTAAACAAAACTGTGGATG-3′	EagI
Map_S84G_-Rv	5′-GCCGAGAAAAGTAATCTGCTCTTGCTTG-3′	
Map_R85A_-Fw	5′-GCCGCAGTAAACAAAACTGTGGATG-3′	EagI
Map_R85A_-Rv	5′-CGATAGAAAAGTAATCTGCTCTTG-3′	
Map_Y140A_-Fw	5′-TTTTAATCAGAAAGTTGATGAGCAG-3′	HindIII
Map_Y140A_-Rv	5′-GCTTTATTGGATTGCAAAAAGTGAC-3′	
Map_C149A_-Fw	5′-GCAAAGGAGTGGATCCCATTACAC-3′	NaeI
Map_C149A_-Rv	5′-CGGCCTGCTCATCAACTTTCTGATTAAAG-3′	
Map_H133A_-Fw	5′-GCTTTTTTGCAATCCAATAAATACTTTAATCAG-3′	AfeI
Map_H133A_-Rv	5′-GCTAATTGATGATTGTGCACAGTTC-3′	
Map_Citro_-Fw	5′-ACGCGTCGACAAGAAGGAGATATACCATGTTTAATCCAACGGCAATGGTCG-3′	SalI
Map_Citro_-Rv	5′-TTTTCGGCCGCTACAGCCTGGTATCCTGCACAC-3′	EagI
Map_EHEC_-Fw	5′-CCGGAATTCATGTTTAGTCCAATGACAATGGC-3′	EcoRI
Map_EHEC_-Rv	5′-TCCCCCGGGCTACAATCGGGTATCCTGTAC-3′	SmaI

Inverse-PCR (primers are listed in Table 2) was used to introduce single-amino-acid substitution in Map (Map_W74A/E78A_, Map_F82A_, Map_S84G_, Map_R85A_, Map_Y140A_, Map_C149A_ and Map_H133A_) using pSA10-Map (Simpson *et al*., 2006) as template DNA. Successful mutagenesis was confirmed by DNA sequencing; the recombinant plasmids are listed in Table 1.

### Tissue culture: transfection and treatment

3T3 Swiss cells (ATCC CCL-92) and Nck mutant mouse embryonic fibroblasts (Bladt *et al*., 2003) were grown at 37°C in a 5% CO_2_ atmosphere with Dubelcco's minimal Eagle medium high glucose supplemented with 10% (v/v) heat-inactivated fetal calf serum (FCS, Sigma) and 2 mM glutamax (Sigma).

3T3 Swiss cells were transfected with pRK5-myc-Rac-1^T17N^, pRK5-myc-Cdc42^T17N^, pRK5-myc-RhoA^T19N^ and pRK5-myc-ROCK (aa 950–1069) constructs (generously provided by N. Lamarche-Vane, McGill University, Canada) whereas Nck mutant mouse embryonic fibroblasts were transfected with CB6-GFP or CB6-GFP-NCK1 (kindly provided by Dr. Michael Way, Cell Motility Laboratory, London Research Institute, Cancer Research UK) by lipofectamine2000 (Invitrogen) according to the manufacturer's recommendations.

SmartPool Cdc42 and RhoA siRNA were obtained from Dharmacon. Swiss 3T3 cells were transfected with 20 nM siRNA or with control siRNA using HiPerFect (Qiagen) as described by the manufacturer. After 48 h, protein levels in total cell lysates were analysed by Western blotting using antibodies against Cdc42 (Upstate), RhoA (Santa Cruz) or β-tubulin (DHSB).

Chemical inhibition of ROCK was obtained by treatment of cells with Y-27632 (Sigma) at a final concentration of 10 μM, 1 h prior to infection.

### Immunofluorescence microscopy

Cells were grown in 24-well cell culture plates to 60–70% confluency. Monolayers were washed twice with PBS and then infected with 0.5 ml of primed bacterial culture at 37°C in a humidified atmosphere containing 5% CO_2_. Monolayers were washed four times with PBS to remove non-adhering bacteria, fixed in 3% paraformaldehyde in PBS for 15 min at room temperature, washed three times with PBS, treated with 50 mM NH_4_Cl for 10 min formaldehyde function neutralization, permeabilized with PBS-0.2% Triton X-100 and blocked with PBS containing 5% BSA. Monolayers were incubated with primary antibodies diluted in PBS–BSA for 1 h. After washing, coverslips were incubated for 45 min with the secondary antibodies diluted in PBS–BSA. Finally, cells were washed three times in PBS, mounted in DAKO mountain medium and examined by conventional epifluorescence microscopy using a Zeiss Axio imager microscope. Deconvolution of the images was done using the AxioVision LE re. Software (Zeiss); the slice of interest was projected to form the new image.

Filopodia were detected with Oregon-green Phaloïdine (Invitrogen) and bacteria were labelled with polyclonal anti-O127 antibody (kindly provided by Dr. Roberto La Ragione, Veterinary Laboratory Agency, UK). Transfected cells expressing myc tag protein were labelled with monoclonal anti-myc (Millipore). Cy2, RRX or Cy-conjugated donkey anti-mouse and donkey anti-rabbit antibodies (Jackson ImmunoResearch) were used as secondary antibodies.

### GST purification

GST–CRIB (Cdc42/Rac-interactive binding) fusion protein was purified as described previously (Haddad *et al*., 2001). Plasmid encoding GST–CNF1 (cytotoxic necrotizing factor 1) was generously provided by J. Bertoglio (U-769 INSERM). *E. coli* strains carrying pGEX plasmids (GE healthcare), pGEX-CRIB and pGEX-CNF1 were grown overnight in LB medium with ampicillin. Subculture were diluted to 1/100 in fresh medium and incubated at 37°C or 25°C (GST–CNF1) until the culture reached an optical density of 0.6 (λ = 600 nm). The 1 mM IPTG was added for 4 h or overnight (GST–CNF1). Bacteria were harvested by centrifugation at 10000 r.p.m. for 20 min at 4°C, and the GST fusion proteins were purified with glutathione-Sepharose (Pharmacia) and dialysed in 0.1 M Tris pH 8, 150 mM NaCl and 1 mM DTT. Protein concentration and purity were determined by BCA assay and SDS page.

### Localization of active Cdc42

Cell are infected and treated as for immunofluoresence. After washing three times with PBS and blocked with BSA-PBS, permeabilized cells were incubated with purified GST or GST–CRIB (1 μg ml^−1^) dissolved in PBS supplemented with BSA. Coverslips were treated as described above for immunofluorescence using rabbit anti-GST antibody (Sigma).

### GST pull-down experiments

For affinity precipitation of Cdc42-GTP cell monolayers in 25 cm^2^ flasks were washed twice with PBS, re-cultured in serum-free medium for 3 h before infection at 37°C in a humidified atmosphere containing 5% CO_2_ with activated EPEC. Cells were washed with cold PBS then lysed in solubilizing buffer containing 50 mM Tris, 1% Triton X-100, 0.5% sodium deoxycholate, 0.1% SDS, 500 mM NaCl, 10 mM MgCl_2_ and cocktail protease inhibitor (Sigma), pH 7.5, for 20 min at 4°C with gentle agitation. Cell lysates were centrifuged to remove insoluble materials. Protein concentration was determined by BC assay protein determination kit (Interchim). Purified GST–CRIB proteins were adsorbed (20 mg) onto glutathione-Sepharose beads (Amersham Pharmacia Biotech). Pre-cleared lysates (500 mg) were rocked with coupled beads for 1 h at 4°C. Beads were washed four times with buffer containing 50 mM Tris, 1% Triton X-100, 150 mM NaCl, 10 mM MgCl_2_ and cocktail protease inhibitor (Sigma), pH 7.5 and then, boiled in 4× Laemmli sample buffer. Bound Cdc42-GTP was analysed by Western blotting.

### Western blot

Proteins were resolved by SDS-PAGE, and gels were transferred to polyvinylidene difluoride membrane (GE Healthcare). Membranes were washed with PBS 0.1% Tween, blocked in TBS (0.1% Tween, 3% BSA, 0.5% gelatin) and probed with specific antibodies overnight at 4°C. Blots were then incubated with horseradish peroxidase-linked secondary antibody (Jackson ImmunoResearch), followed by ECL plus assay, according to the manufacturer's instructions (GE Healthcare). Chemiluminescences were detecting using a LAS 3000 Fugi imager. Western blots were then quantified with scion image software. Polyclonal anti-Cdc42 (Upstate) was used to detect Cdc42.

### Statistic analysis

All experiments were conducted in triplicate and repeated at least three times. Results were expressed as means ± standard deviation. The statistical significance was determined by Student's *t*-test, and *P* < 0.05 was considered to be significant.
